# Including probe-level uncertainty in model-based gene expression clustering

**DOI:** 10.1186/1471-2105-8-98

**Published:** 2007-03-21

**Authors:** Xuejun Liu, Kevin K Lin, Bogi Andersen, Magnus Rattray

**Affiliations:** 1 College of Information Science and Technology, Nanjing University of Aeronautics and Astronautics, 29 Yudao Street, Nanjing 210016, China; 2 Departments of Biological Chemistry and Medicine, Institute for Genomics and Bioinformatics, University of California, Irvine, CA 92697, USA; 3 School of Computer Science, University of Manchester, Kilburn Building, Oxford Road, Manchester M13 9PL, UK

## Abstract

**Background:**

Clustering is an important analysis performed on microarray gene expression data since it groups genes which have similar expression patterns and enables the exploration of unknown gene functions. Microarray experiments are associated with many sources of experimental and biological variation and the resulting gene expression data are therefore very noisy. Many heuristic and model-based clustering approaches have been developed to cluster this noisy data. However, few of them include consideration of probe-level measurement error which provides rich information about technical variability.

**Results:**

We augment a standard model-based clustering method to incorporate probe-level measurement error. Using probe-level measurements from a recently developed Affymetrix probe-level model, multi-mgMOS, we include the probe-level measurement error directly into the standard Gaussian mixture model. Our augmented model is shown to provide improved clustering performance on simulated datasets and a real mouse time-course dataset.

**Conclusion:**

The performance of model-based clustering of gene expression data is improved by including probe-level measurement error and more biologically meaningful clustering results are obtained.

## Background

Microarrays [1,2] are routinely used for the quantitative measurement of gene expression levels on a genome-wide scale. Microarray experiments are complicated multiple step procedures and variability can be introduced in every step, so that the resulting data are often very noisy, especially for weakly expressed genes. Appropriate statistical analysis of this noisy data is very important in order to obtain meaningful biological information [3,4]. The analysis of microarray data is usually performed in multiple stages, including probe-level analysis, normalisation and higher level analyses. The aim of the probe-level analysis is to obtain reliable gene expression measurements from the image data. Various higher level analyses, such as detecting differential gene expression or clustering, can then be carried out depending on the biological aims of the experiment.

Unsupervised clustering is the most frequently used approach for exploring gene function. By clustering, a huge number of genes can be organised into a much smaller number of categories according to their shared expression patterns. It is hoped that these shared patterns reflect similar function or common transcriptional regulation. Exploring and studying the obtained gene clusters is an important way to infer the function of uncharacterised genes from other known genes in the same cluster. There are many unsupervised algorithms which have been used to cluster gene expression data, including the most popular hierarchical clustering [5] and *k*-means [6], which are based on similarity measures, and self-organising maps [7]. Most of these conventional algorithms are largely heuristically motivated. They are easily implemented and their application is usually computationally efficient. However, these methods lack the capability to deal in a principled way with the experimental variability in the gene expression data. Furthermore, there is no formal way to determine the number of clusters with these algorithms. It is hard to say which one is generally better than the others [8]. Probabilistic models provide a principled alternative to these conventional methods. In particular, model-based approaches have been proposed as useful methods for clustering gene expression data in a probabilistic way [9-12]. By using a probabilistic model, the experimental noise can be included explicitly in the model and estimated from the data, making this approach more robust to noise. There are also useful and principled model selection methods that can be used to determine the optimal number of clusters. The advantages of model-based probabilistic approaches over heuristic methods are already well established [10].

Affymetrix arrays contain multiple probes for each target gene and this internal replication can be used to obtain an estimate of the technical measurement error associated with each gene expression measurement [13-17]. This source of error is especially significant for weakly expressed genes. The recently developed model, multi-mgMOS [18], provides accurate gene expression measurements along with the associated uncertainty in this measurement. It has been shown that the probe-level measurement error obtained from multi-mgMOS can be propagated through a downstream probabilistic analysis, thereby improving the performance of the analysis [16,17]. Existing model-based clustering methods do not consider this probe-level measurement error and they therefore discard this rich source of information about variability. Although standard model-based clustering methods are relatively robust to noise, very noisy measurements can still have a detrimental effect on these clustering methods, resulting in poor performance and many biologically irrelevant clusters. In this paper, we aim to include information about probe-level measurement error into the standard Gaussian mixture model in order to improve performance compared to standard model-based clustering. Our augmented Gaussian mixture clustering model is called PUMA-CLUST (Propagating Uncertainty in Microarray Analysis – CLUSTering) and has been implemented in the R-package *pumaclust *which is available from [19].

## Results and discussion

We examine the performance of the extended Gaussian mixture model on two simulated datasets and a real-world mouse time-course dataset [12]. The simulated datasets are generated to reflect the noise commonly seen in real microarray experiments. The extended mixture model is compared with the standard Gaussian mixture models implemented in MCLUST [20], which includes all variants of standard Gaussian mixture models in terms of the representation of the covariance matrix. However, these models do not take the probe-level measurement error into consideration.

The performance of different clustering methods on datasets with known structures can be evaluated by using the adjusted Rand index [21,22]. The adjusted Rand index measures the similarity of two clusterings on a dataset and it is widely used by the clustering research community [10,23-25]. The adjusted Rand index lies between 0 and 1, and is calculated based on whether pairs are placed in the same or different clusters in two partitions with a higher value meaning better agreement between two clusterings. For the simulated datasets, since the true structure of the data is known, we use the adjusted Rand index to evaluate the different partitioning ability of the extended mixture model which incorporates the probe-level measurement error and the standard mixture model. For the real mouse time-course dataset, gene ontology (GO) enrichment analysis is used to compare the performance of the two clustering methods.

### Clustering on simulated data sets

#### Simulated periodic data

Periodic patterns are often observed in real-world time-course microarray data [12,26]. However, the true structure of the real datasets is unavailable. We generate simulated periodic data and include noise with magnitude estimated from real microarray data. Similar to the methods used by [23] and [25], the simulated data is generated by the following four steps.

At the first step, the logged gene expression within each known group is generated. There are six groups and 600 genes in the dataset. Each group has 100 genes. The first four groups have a periodic sine pattern. The expression of gene *i *in group *q*, *q *= 1, 2, 3, 4, is generated by

*x*_*qij *_= *A*_*i *_sin(2*πj*/10 - *πq/*2)* + S*,     (1)

where *j *= 1, 2,..., *J *and *J *is the number of conditions or time points. *A*_*i *_is a random scaling factor which is sampled from U(0, 7), where U represents the uniform distribution. *S *is a shifting factor which is set as 7. This assignment of *A*_*i *_and *S *is to make the gene expression level lie between 0 and 14 which is the normal range of the logged gene expression level from real Affymetrix datasets. The gene expression levels of group 5 and group 6 are generated by linear functions

*x*_*qij *_= *jA*_*qi*_/*J *and *x*_*qij *_= -*jA*_*qi*_/*J *+ *S*,     (2)

respectively, where *A*_*qi *_is sampled from *U *(0,14) and *S *= 14 when *q *= 6 so as to ensure that the simulated expression level lies within the accepted logged expression range.

**Figure 1:**
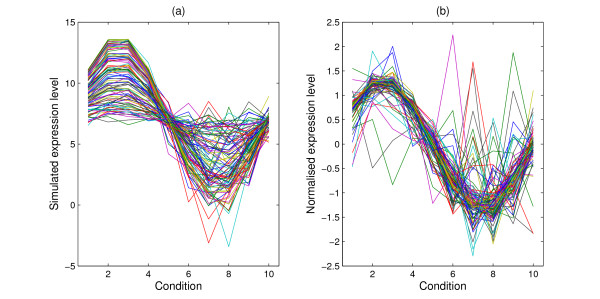
**Simulated expression profiles**. Simulated expression profiles for one group under 10 conditions. (a) are the raw data on a log scale and (b) are the normalised profiles with zero mean and standard deviation one.

The simulated data from the first step follows perfectly the same sine wave within the same group except for a different magnitude. However, in practice there is biological and technical noise in the experiment distorting the true sine wave. At the second step, the real mouse dataset (described in the next section) is used to obtain an estimate of the combined noise from biological and technical sources which is related to the variance of observed gene expression level from replicated experiments. The mouse dataset has three or four replicates for each condition. Using the gene expression summaries from MAS 5.0 [27] which is the standard software provided by Affymetrix, an estimate of the combined technical and biological noise can be obtained from Cyber-T [28]. Cyber-T is a Bayesian hierarchical model which calculates the variance between replicates using point estimates of gene expression level from each replicate. Since the variance has a dependence on gene expression level, the combined noise, σqij2
 MathType@MTEF@5@5@+=feaafiart1ev1aaatCvAUfKttLearuWrP9MDH5MBPbIqV92AaeXatLxBI9gBaebbnrfifHhDYfgasaacH8akY=wiFfYdH8Gipec8Eeeu0xXdbba9frFj0=OqFfea0dXdd9vqai=hGuQ8kuc9pgc9s8qqaq=dirpe0xb9q8qiLsFr0=vr0=vr0dc8meaabaqaciaacaGaaeqabaqabeGadaaakeaaiiGacqWFdpWCdaqhaaWcbaGaemyCaeNaemyAaKMaemOAaOgabaGaeGOmaidaaaaa@33B8@, is sampled from a subset of variances calculated from Cyber-T whose corresponding expression levels are close to *x*_*qij*_. Thus, the final simulated expression level, x^
 MathType@MTEF@5@5@+=feaafiart1ev1aaatCvAUfKttLearuWrP9MDH5MBPbIqV92AaeXatLxBI9gBaebbnrfifHhDYfgasaacH8akY=wiFfYdH8Gipec8Eeeu0xXdbba9frFj0=OqFfea0dXdd9vqai=hGuQ8kuc9pgc9s8qqaq=dirpe0xb9q8qiLsFr0=vr0=vr0dc8meaabaqaciaacaGaaeqabaqabeGadaaakeaacuWG4baEgaqcaaaa@2E35@_*qij*_, is

x^
 MathType@MTEF@5@5@+=feaafiart1ev1aaatCvAUfKttLearuWrP9MDH5MBPbIqV92AaeXatLxBI9gBaebbnrfifHhDYfgasaacH8akY=wiFfYdH8Gipec8Eeeu0xXdbba9frFj0=OqFfea0dXdd9vqai=hGuQ8kuc9pgc9s8qqaq=dirpe0xb9q8qiLsFr0=vr0=vr0dc8meaabaqaciaacaGaaeqabaqabeGadaaakeaacuWG4baEgaqcaaaa@2E35@_*qij *_= *x*_*qij *_+ *ε*_*qij*_,     (3)

where *ε*_*qij *_is drawn from N
 MathType@MTEF@5@5@+=feaafiart1ev1aaatCvAUfKttLearuWrP9MDH5MBPbIqV92AaeXatLxBI9gBamrtHrhAL1wy0L2yHvtyaeHbnfgDOvwBHrxAJfwnaebbnrfifHhDYfgasaacH8akY=wiFfYdH8Gipec8Eeeu0xXdbba9frFj0=OqFfea0dXdd9vqai=hGuQ8kuc9pgc9s8qqaq=dirpe0xb9q8qiLsFr0=vr0=vr0dc8meaabaqaciaacaGaaeqabaWaaeGaeaaakeaaimaacqWFneVtaaa@383B@(0, σqij2
 MathType@MTEF@5@5@+=feaafiart1ev1aaatCvAUfKttLearuWrP9MDH5MBPbIqV92AaeXatLxBI9gBaebbnrfifHhDYfgasaacH8akY=wiFfYdH8Gipec8Eeeu0xXdbba9frFj0=OqFfea0dXdd9vqai=hGuQ8kuc9pgc9s8qqaq=dirpe0xb9q8qiLsFr0=vr0=vr0dc8meaabaqaciaacaGaaeqabaqabeGadaaakeaaiiGacqWFdpWCdaqhaaWcbaGaemyCaeNaemyAaKMaemOAaOgabaGaeGOmaidaaaaa@33B8@). When *J *= 10, the simulated expression level for group three is shown in Figure 1(a). It can be seen that there is more noise for the lower expressed genes than the highly expressed ones, which is a common feature of real datasets.

**Figure 2:**
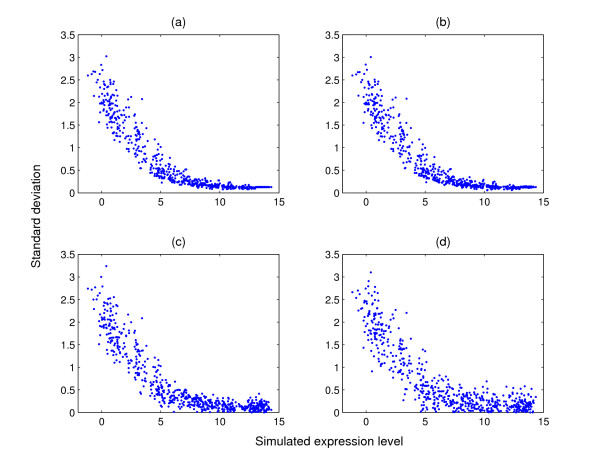
**Standard deviation against the simulated gene expression level**. Scatter plots of standard deviation against the simulated gene expression level. The standard deviation in (a) is sampled from the multi-mgMOS results obtained from the mouse dataset. The standard deviation is randomly changed by adding a noise drawn from (b) N
 MathType@MTEF@5@5@+=feaafiart1ev1aaatCvAUfKttLearuWrP9MDH5MBPbIqV92AaeXatLxBI9gBamrtHrhAL1wy0L2yHvtyaeHbnfgDOvwBHrxAJfwnaebbnrfifHhDYfgasaacH8akY=wiFfYdH8Gipec8Eeeu0xXdbba9frFj0=OqFfea0dXdd9vqai=hGuQ8kuc9pgc9s8qqaq=dirpe0xb9q8qiLsFr0=vr0=vr0dc8meaabaqaciaacaGaaeqabaWaaeGaeaaakeaaimaacqWFneVtaaa@383B@(0, 0.01), (c) N
 MathType@MTEF@5@5@+=feaafiart1ev1aaatCvAUfKttLearuWrP9MDH5MBPbIqV92AaeXatLxBI9gBamrtHrhAL1wy0L2yHvtyaeHbnfgDOvwBHrxAJfwnaebbnrfifHhDYfgasaacH8akY=wiFfYdH8Gipec8Eeeu0xXdbba9frFj0=OqFfea0dXdd9vqai=hGuQ8kuc9pgc9s8qqaq=dirpe0xb9q8qiLsFr0=vr0=vr0dc8meaabaqaciaacaGaaeqabaWaaeGaeaaakeaaimaacqWFneVtaaa@383B@(0, 0.1) and (d) N
 MathType@MTEF@5@5@+=feaafiart1ev1aaatCvAUfKttLearuWrP9MDH5MBPbIqV92AaeXatLxBI9gBamrtHrhAL1wy0L2yHvtyaeHbnfgDOvwBHrxAJfwnaebbnrfifHhDYfgasaacH8akY=wiFfYdH8Gipec8Eeeu0xXdbba9frFj0=OqFfea0dXdd9vqai=hGuQ8kuc9pgc9s8qqaq=dirpe0xb9q8qiLsFr0=vr0=vr0dc8meaabaqaciaacaGaaeqabaWaaeGaeaaakeaaimaacqWFneVtaaa@383B@(0, 0.2).

At the third step, in order to show the clustering improvement by including probe-level measurement error, we sample the corresponding probe-level variance of the simulated expression level from the real mouse dataset processed by multi-mgMOS. Similar to the second step, since the measurement error has a dependence on the gene expression level, the standard deviation for each simulated expression value, σ^
 MathType@MTEF@5@5@+=feaafiart1ev1aaatCvAUfKttLearuWrP9MDH5MBPbIqV92AaeXatLxBI9gBaebbnrfifHhDYfgasaacH8akY=wiFfYdH8Gipec8Eeeu0xXdbba9frFj0=OqFfea0dXdd9vqai=hGuQ8kuc9pgc9s8qqaq=dirpe0xb9q8qiLsFr0=vr0=vr0dc8meaabaqaciaacaGaaeqabaqabeGadaaakeaaiiGacuWFdpWCgaqcaaaa@2E86@_*qij *_is sampled from a subset of standard deviation calculated from multi-mgMOS whose corresponding expression levels are close to x^
 MathType@MTEF@5@5@+=feaafiart1ev1aaatCvAUfKttLearuWrP9MDH5MBPbIqV92AaeXatLxBI9gBaebbnrfifHhDYfgasaacH8akY=wiFfYdH8Gipec8Eeeu0xXdbba9frFj0=OqFfea0dXdd9vqai=hGuQ8kuc9pgc9s8qqaq=dirpe0xb9q8qiLsFr0=vr0=vr0dc8meaabaqaciaacaGaaeqabaqabeGadaaakeaacuWG4baEgaqcaaaa@2E35@_*qij*_. Figure 2(a) shows the scatter plot of the sampled standard deviation against the simulated expression level for one randomly selected condition. It can be seen that the variance of the measured gene expression for the weakly expressed genes is generally larger than that for the highly expressed genes as is commonly observed in real datasets. This is consistent with the plot in Figure 1(a). At the final step, we normalise the simulated expression level for each gene over all conditions by subtracting the mean expression level and dividing by the standard deviation such that the profile of each gene has zero mean and standard deviation one. The simulated standard deviation is also divided by the standard deviation of the expression level to determine the corresponding measurement error of the normalised data. The normalised profile is shown in Figure 1(b) when *J *= 10.

**Figure 3:**
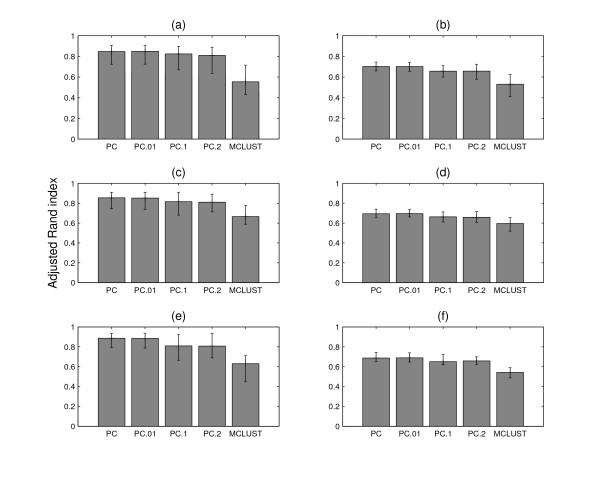
**Average adjusted Rand index**. The average adjusted Rand index of the clustering results from PUMA-CLUST and MCLUST on the simulated data. The first column is for the six group dataset and the second column is for the seven group dataset with one noise group added. The upper panel shows results on datasets with 10 conditions, the middle panel is for 20 conditions and the lower panel is for 30 conditions. PC represents PUMA-CLUST results on the original simulated data. PC.01, PC.1 and PC.2 represent the PUMA-CLUST results on the datasets with added noise drawn from N
 MathType@MTEF@5@5@+=feaafiart1ev1aaatCvAUfKttLearuWrP9MDH5MBPbIqV92AaeXatLxBI9gBamrtHrhAL1wy0L2yHvtyaeHbnfgDOvwBHrxAJfwnaebbnrfifHhDYfgasaacH8akY=wiFfYdH8Gipec8Eeeu0xXdbba9frFj0=OqFfea0dXdd9vqai=hGuQ8kuc9pgc9s8qqaq=dirpe0xb9q8qiLsFr0=vr0=vr0dc8meaabaqaciaacaGaaeqabaWaaeGaeaaakeaaimaacqWFneVtaaa@383B@(0, 0.01), N
 MathType@MTEF@5@5@+=feaafiart1ev1aaatCvAUfKttLearuWrP9MDH5MBPbIqV92AaeXatLxBI9gBamrtHrhAL1wy0L2yHvtyaeHbnfgDOvwBHrxAJfwnaebbnrfifHhDYfgasaacH8akY=wiFfYdH8Gipec8Eeeu0xXdbba9frFj0=OqFfea0dXdd9vqai=hGuQ8kuc9pgc9s8qqaq=dirpe0xb9q8qiLsFr0=vr0=vr0dc8meaabaqaciaacaGaaeqabaWaaeGaeaaakeaaimaacqWFneVtaaa@383B@(0, 0.1) and N
 MathType@MTEF@5@5@+=feaafiart1ev1aaatCvAUfKttLearuWrP9MDH5MBPbIqV92AaeXatLxBI9gBamrtHrhAL1wy0L2yHvtyaeHbnfgDOvwBHrxAJfwnaebbnrfifHhDYfgasaacH8akY=wiFfYdH8Gipec8Eeeu0xXdbba9frFj0=OqFfea0dXdd9vqai=hGuQ8kuc9pgc9s8qqaq=dirpe0xb9q8qiLsFr0=vr0=vr0dc8meaabaqaciaacaGaaeqabaWaaeGaeaaakeaaimaacqWFneVtaaa@383B@(0, 0.2) respectively. The average adjusted Rand index is calculated over 10 simulated datasets for each plot and the range of the adjusted Rand index of each case is shown by error bars.

Since the true partition of the simulated dataset is known, the agreement of the clustering results from different methods with the true partition can be assessed by the adjusted Rand index. The true number of groups, six, is selected for both MCLUST and PUMA-CLUST. Three sets of datasets are generated to evaluate the different performance of PUMA-CLUST and MCLUST with number of conditions 10, 20 and 30. For each set, 10 random simulated datasets are generated. The average adjusted Rand index from PUMA-CLUST and MCLUST are shown in the first column of Figure 3. For the three sets of simulated datasets, PUMA-CLUST results in markedly better performance compared with MCLUST and the *p*-values of a paired t-test, shown in Table 1, indicate that the difference in performance is highly significant.

**Table 1:** *P*-values obtained from a paired t-test of adjusted Rand index from MCLUST and PUMA-CLUST. A paired t-test is performed for MCLUST and each of PUMA-CLUST results. The 10 simulated datasets in Figure 3 are used for each test. PC represents PUMA-CLUST results on the original simulated data. PC.01, PC.1 and PC.2 represent the PUMA-CLUST results on the datasets with added noise drawn from N
 MathType@MTEF@5@5@+=feaafiart1ev1aaatCvAUfKttLearuWrP9MDH5MBPbIqV92AaeXatLxBI9gBamrtHrhAL1wy0L2yHvtyaeHbnfgDOvwBHrxAJfwnaebbnrfifHhDYfgasaacH8akY=wiFfYdH8Gipec8Eeeu0xXdbba9frFj0=OqFfea0dXdd9vqai=hGuQ8kuc9pgc9s8qqaq=dirpe0xb9q8qiLsFr0=vr0=vr0dc8meaabaqaciaacaGaaeqabaWaaeGaeaaakeaaimaacqWFneVtaaa@383B@(0, 0.01), N
 MathType@MTEF@5@5@+=feaafiart1ev1aaatCvAUfKttLearuWrP9MDH5MBPbIqV92AaeXatLxBI9gBamrtHrhAL1wy0L2yHvtyaeHbnfgDOvwBHrxAJfwnaebbnrfifHhDYfgasaacH8akY=wiFfYdH8Gipec8Eeeu0xXdbba9frFj0=OqFfea0dXdd9vqai=hGuQ8kuc9pgc9s8qqaq=dirpe0xb9q8qiLsFr0=vr0=vr0dc8meaabaqaciaacaGaaeqabaWaaeGaeaaakeaaimaacqWFneVtaaa@383B@(0, 0.1) and N
 MathType@MTEF@5@5@+=feaafiart1ev1aaatCvAUfKttLearuWrP9MDH5MBPbIqV92AaeXatLxBI9gBamrtHrhAL1wy0L2yHvtyaeHbnfgDOvwBHrxAJfwnaebbnrfifHhDYfgasaacH8akY=wiFfYdH8Gipec8Eeeu0xXdbba9frFj0=OqFfea0dXdd9vqai=hGuQ8kuc9pgc9s8qqaq=dirpe0xb9q8qiLsFr0=vr0=vr0dc8meaabaqaciaacaGaaeqabaWaaeGaeaaakeaaimaacqWFneVtaaa@383B@(0, 0.2) respectively.

No of conditions	6 groups				7 groups			
	
	PC	PC.01	PC.1	PC.2	PC	PC.01	PC.1	PC.2
10	1.10e-8	9.37e-8	5.90e-8	5.67e-7	5.67e-9	7.77e-9	3.87e-7	5.87e-6
20	2.39e-8	1.80e-8	2.30e-7	4.22e-7	4.03e-9	4.10e-9	1.13e-7	8.56e-8
30	3.54e-7	1.38e-6	2.99e-6	5.00e-6	9.96e-7	4.34e-7	1.14e-7	3.75e-6

#### Including a noise group

In a real-world microarray dataset, there are usually a certain fraction of genes whose expression levels are indistinguishable from random noise. These genes do not belong to any pattern group in the dataset [25].

To assess the performance of PUMA-CLUST on this kind of dataset, we add a group of random noise genes into the previously simulated datasets. The first generating step of the gene expression level for group seven is

*x*_*qij *_= *A*_*qi*_,     (4)

where *A*_*qi *_is sampled from *U*(0,14). The following steps of the simulation are the same as those for the former six groups. Three sets of simulated datasets with 10 randomly generated datasets for each set are also sampled and the average adjusted Rand index for three cases with 10, 20, and 30 conditions are shown in the second column of Figure 3. The number of groups for both MCLUST and PUMA-CLUST is assigned to seven. From the three plots it can be seen that the performance of the clustering from both PUMA-CLUST and MCLUST decreases with the inclusion of the noise group, but PUMA-CLUST still outperforms MCLUST over all three noise levels with the three different data dimensions. The *p*-values in Table 1 indicate that the improvement is statistically significant.

#### Testing the robustness to misspecified technical variance

The probe-level variance in the simulated datasets generated above is sampled from multi-mgMOS results from the real mouse dataset. When applying PUMA-CLUST it was assumed that the level of noise is known, but in practice it would be estimated using multi-mgMOS. We would like to test robustness to errors in estimating the measurement error variance. We therefore add some noise to the sampled standard deviation, σ^
 MathType@MTEF@5@5@+=feaafiart1ev1aaatCvAUfKttLearuWrP9MDH5MBPbIqV92AaeXatLxBI9gBaebbnrfifHhDYfgasaacH8akY=wiFfYdH8Gipec8Eeeu0xXdbba9frFj0=OqFfea0dXdd9vqai=hGuQ8kuc9pgc9s8qqaq=dirpe0xb9q8qiLsFr0=vr0=vr0dc8meaabaqaciaacaGaaeqabaqabeGadaaakeaaiiGacuWFdpWCgaqcaaaa@2E86@_*qij*_, to simulate the error made in estimating this quantity. For the six-group and seven-group datasets, three kinds of random noise are added by sampling from N
 MathType@MTEF@5@5@+=feaafiart1ev1aaatCvAUfKttLearuWrP9MDH5MBPbIqV92AaeXatLxBI9gBamrtHrhAL1wy0L2yHvtyaeHbnfgDOvwBHrxAJfwnaebbnrfifHhDYfgasaacH8akY=wiFfYdH8Gipec8Eeeu0xXdbba9frFj0=OqFfea0dXdd9vqai=hGuQ8kuc9pgc9s8qqaq=dirpe0xb9q8qiLsFr0=vr0=vr0dc8meaabaqaciaacaGaaeqabaWaaeGaeaaakeaaimaacqWFneVtaaa@383B@(0, 0.01), N
 MathType@MTEF@5@5@+=feaafiart1ev1aaatCvAUfKttLearuWrP9MDH5MBPbIqV92AaeXatLxBI9gBamrtHrhAL1wy0L2yHvtyaeHbnfgDOvwBHrxAJfwnaebbnrfifHhDYfgasaacH8akY=wiFfYdH8Gipec8Eeeu0xXdbba9frFj0=OqFfea0dXdd9vqai=hGuQ8kuc9pgc9s8qqaq=dirpe0xb9q8qiLsFr0=vr0=vr0dc8meaabaqaciaacaGaaeqabaWaaeGaeaaakeaaimaacqWFneVtaaa@383B@(0, 0.1) and N
 MathType@MTEF@5@5@+=feaafiart1ev1aaatCvAUfKttLearuWrP9MDH5MBPbIqV92AaeXatLxBI9gBamrtHrhAL1wy0L2yHvtyaeHbnfgDOvwBHrxAJfwnaebbnrfifHhDYfgasaacH8akY=wiFfYdH8Gipec8Eeeu0xXdbba9frFj0=OqFfea0dXdd9vqai=hGuQ8kuc9pgc9s8qqaq=dirpe0xb9q8qiLsFr0=vr0=vr0dc8meaabaqaciaacaGaaeqabaWaaeGaeaaakeaaimaacqWFneVtaaa@383B@(0, 0.2). The scatter plots of the error-added standard deviation against the simulated gene expression are shown in Figure 2(b)–(d). Figure 3 gives the average adjusted Rand index of the clustering results from PUMA-CLUST on the error-added standard deviation for various cases. In the case of PC.01, the added noise is quite small so that the clustering results of PC.01 are very close to the clustering results on the original simulated data. As the added noise variance increases, the performance of PUMA-CLUST decreases. The *p*-values in Table 1 mostly increase when larger noise is added to the variances but all *p*-values remain small and demonstrate a significant improvement for PUMA-CLUST over MCLUST. These results demonstrate that clustering is most accurate when the measurement error variance is known, but that the method is robust to errors in the estimate of the measurement error.

### Clustering on a real mouse time-course dataset

The improved performance of the new model, PUMA-CLUST, over the standard Gaussian mixture model on simulated datasets was shown in the previous section. Here, we evaluate the performance of PUMA-CLUST on a real mouse dataset showing periodic behavior [12] by comparing with the results of the standard mixture model implemented in MCLUST.

This time-course dataset profiles the gene expression changes during the hair growth cycle, which is synchronised for the first two cycles following birth. After two cycles the hair growth cycle becomes progressively unsynchronised. Lin et al. use Affymetrix MG-U74Av2 microarray chips to profile mRNA expression in mouse back skin from eight representative time points in order to discover regulators in hair-follicle morphogenesis and cycling [12]. The microarray dataset utilised a total of 25 chips with each time point consisting of three or four replicates. The first five time points (day 1, 6, 14, 17 and 23) cover the first synchronised cycle and the last three time points (week 9, month 5 and year 1) belong to the asynchronous cycles. They identified 2,461 potential hair cycle-associated genes using a *F *test comparing synchronous and asynchronous time points. This dataset is available at [29].

We apply both PUMA-CLUST and MCLUST clustering over the first five time points which belong to the synchronised cycle and includes 15 chips. For MCLUST the raw mouse dataset is processed using the popular probe-level method GCRMA [30]. For PUMA-CLUST the raw data is processed by multi-mgMOS. We also applied MCLUST to MAS5.0 and multi-mgMOS gene expression measurements and the performance was found to be similar to the results presented here using GCRMA.

**Figure 4:**
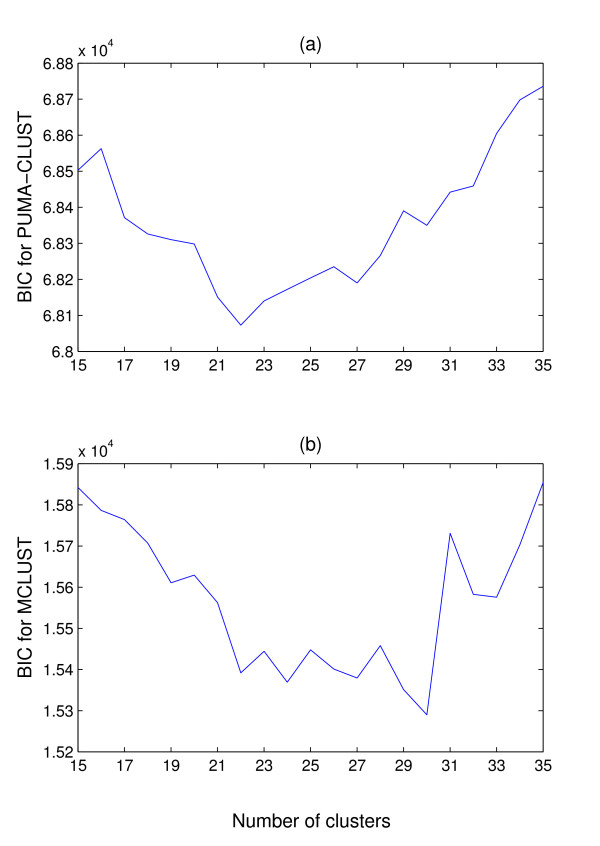
**BIC for PUMA-CLUST and MCLUST**. BIC for (a) PUMA-CLUST and (b) MCLUST against the number of mixture components on the 2,461 potential hair growth-associated genes from the mouse time-course dataset. PUMA-CLUST obtains the minimum BIC at K = 22 and MCLUST obtains the minimum at K = 30.

**Figure 5:**
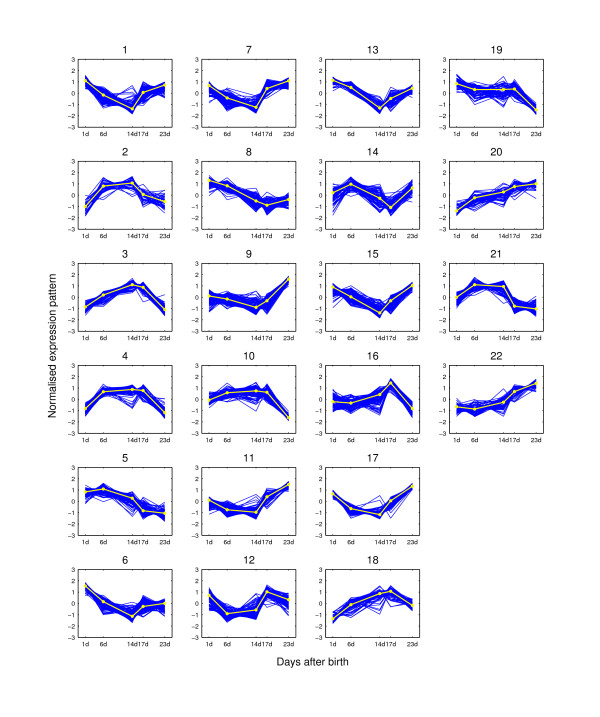
**Expression pattern clusters from PUMA-CLUST when K = 22**. The clusters are for the 2,461 potential hair cycle-associated genes of the mouse time-course dataset when K = 22. The expression pattern for each probe-set is shown as dark lines for five time points. The light line on each plot is the clustering center for each group. At each time point, the expression value is the average of the three replicated measurements.

**Figure 6:**
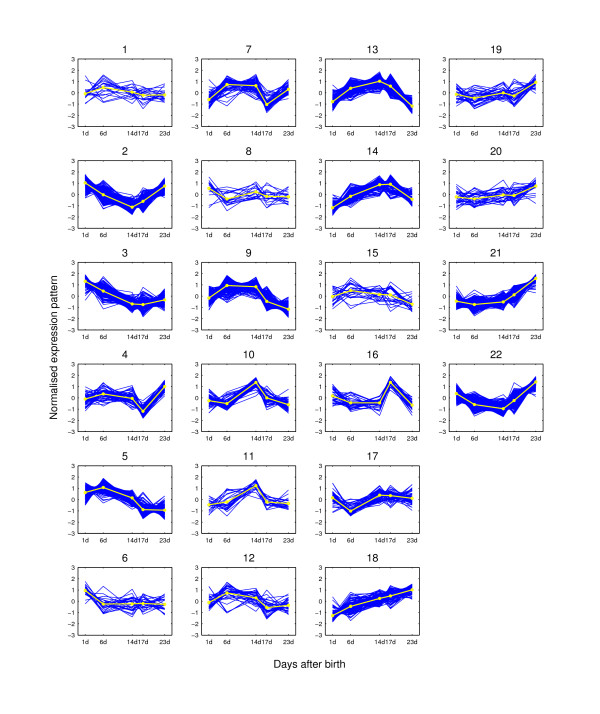
**Expression pattern clusters from MCLUST when K = 22**. The clusters are for the 2,461 potential hair cycle-associated genes of the mouse time-course dataset when K = 22. The expression pattern for each probe-set is shown as dark lines for five time points. The light line on each plot is the clustering center for each group. At each time point, the expression value is the average of the three replicated measurements.

**Figure 7:**
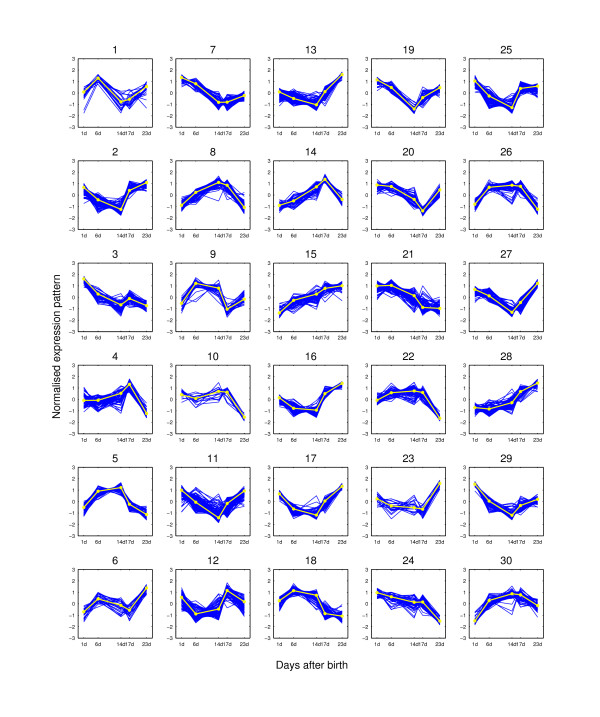
**Expression pattern clusters from PUMA-CLUST when K = 30**. The clusters are for the 2,461 potential hair-growth-associated genes of the mouse time-course dataset when K = 30. The expression pattern for each probe-set is shown as dark lines for five time points. The light line on each plot is the clustering center for each group. At each time point, the expression value is the average of the three replicated measurements.

**Figure 8:**
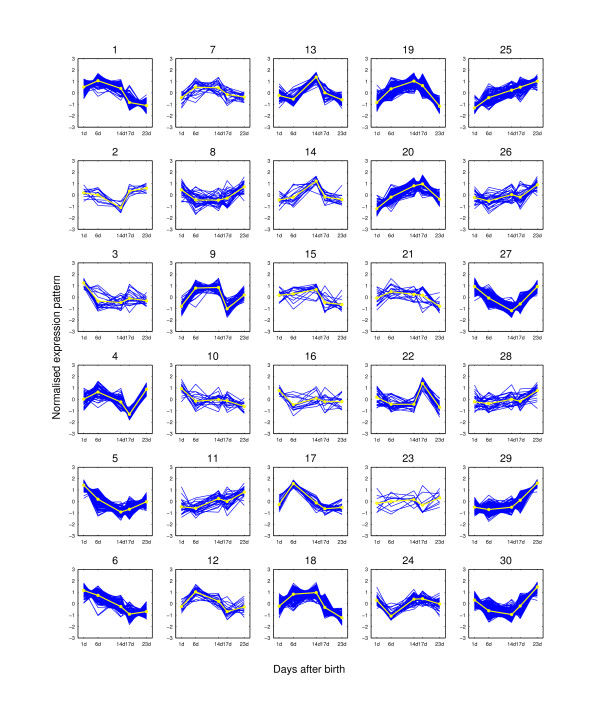
**Expression pattern clusters from MCLUST when K = 30**. The clusters are for the 2,461 potential hair-growth-associated genes of the mouse time-course dataset when K = 30. The expression pattern for each probe-set is shown as dark lines for five time points. The light line on each plot is the clustering center for each group. At each time point, the expression value is the average of the three replicated measurements.

The clustering is performed on the 2,461 potential hair cycle-associated genes. The obtained expression level for each probe-set from both probe-level methods are normalised to have zero mean and standard deviation one. The Bayesian Information Criterion (BIC [31]) is used to determine the number of clusters. The calculated BIC for various numbers of clusters is shown in Figure 4. It can be seen that the optimal BIC for PUMA-CLUST is obtained at K = 22 and the optimal BIC for MCLUST is obtained at K = 30. In both cases, MCLUST converges to the model having the same full rank covariance matrix for each component (the 'EEE' model [32]). In order to make the different clustering methods comparable, the number of clusters for each method should be the same. Therefore, the 22-cluster and the 30-cluster cases are compared separately. The 22 clusters obtained from PUMA-CLUST and MCLUST are shown in Figure 5 and Figure 6 respectively, and the 30 clusters obtained are shown in Figure 7 and Figure 8, respectively. For visualisation, the average expression level at each time point over replicates is shown for both the gene profile and the cluster center.

To assess whether biologically relevant clusters are created using the two methods, we systematically performed GO annotation enrichment analysis for the individual clusters using DAVID 2006 (The Database for Annotation, Visualization and Integrated Discovery, [33]). The GO enrichment analysis allows the direct assessment of the biological significance for gene clusters found based on the enrichment of genes belonging to a specific GO functional category. The enrichment calculation performed in DAVID is a modified Fisher Exact test. The resulting p-value shows the biological significance for gene clusters. Based on our experience, GO Biological Process term level 5 gives more precise category definitions which are useful in further biological interpretations. Therefore, a meaningful GO enrichment analysis is to examine enriched categories of GO Biological Process at term level 5 and to select an enrichment cutoff at a conventional p-value of 0.05.

**Figure 9:**
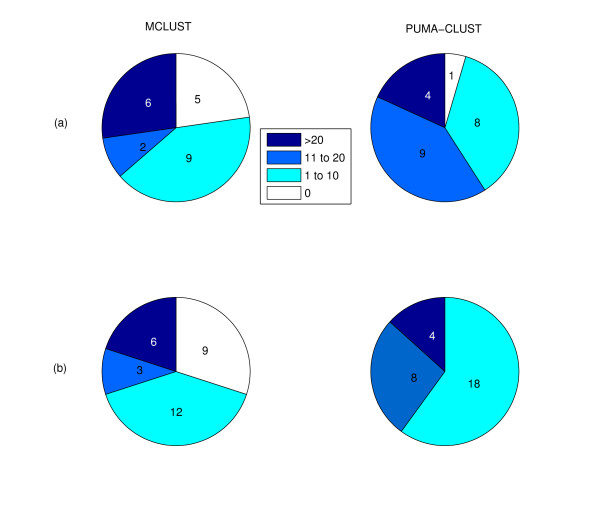
**Comparison of the number of clusters found with the indicated ranges of enriched GO categories for MCLUST and PUMA-CLUST clusters**. Comparison of the number of clusters found with the indicated ranges of enriched categories for MCLUST and PUMA-CLUST clusters using (a) 22 clusters and (b) 30 clusters. For both comparisons, the enriched categories were found using GO Biological Process term level 5, enrichment cutoff at p-value of 0.05, and mouse (*Mus Musculus*) as the population background.

**Figure 10:**
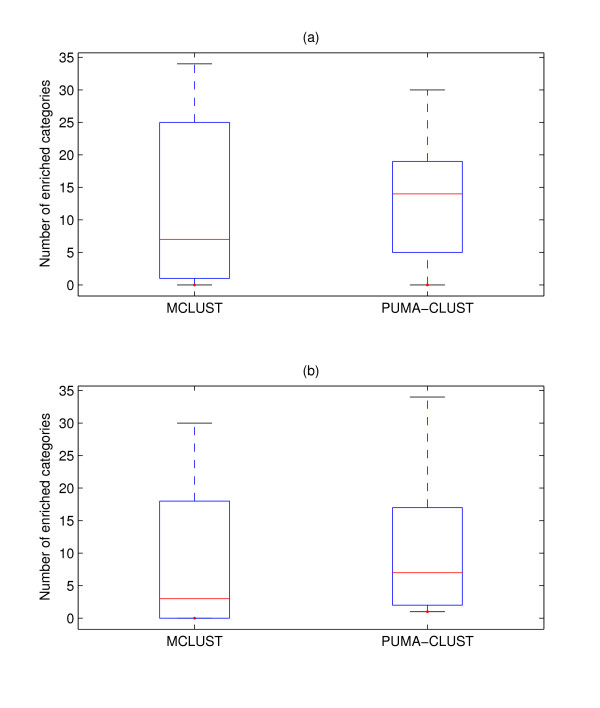
**Boxplot of the number of enriched categories for MCLUST and PUMA-CLUST clusters**. Boxplot of the number of enriched categories for MCLUST and PUMA-CLUST clusters using (a) 22 clusters and (b) 30 clusters. The boxes show the lower quartile, median, and upper quartile values. The dotted lines show the extent of the rest of the data. The number of enriched categories for MCLUST has larger variance than that for PUMA-CLUST.

We found that for the 22-cluster results from the two methods PUMA-CLUST produced more clusters (21 of 22) with at least one enriched GO category in comparison to MCLUST (17 of 22), as shown in Figure 9(a). A visual inspection of these MCLUST clusters without an enriched GO category indicates that four out of five of these clusters (Cluster #1,6,8,15 in Figure 6) contain heterogeneous temporal expression profiles (i.e. not tightly clustered). Since the number of enriched GO categories found varies greatly among clusters (shown in Figure 10(a)), the average number (13.1) of enriched categories among the 22 PUMA-CLUST clusters is only slightly greater than the average among the MCLUST clusters (11.5). A more meaningful indicator of the distribution differences is the median number of enriched categories in PUMA-CLUST clusters (14) and MCLUST clusters (7). The same enrichment analysis method was repeated using the 30 clusters for both methods, and the results still clearly indicate that the PUMA-CLUST method results in more biologically meaningful clusters than the MCLUST method. Using 30 clusters, all clusters generated by PUMA-CLUST have at least one enriched GO category, in comparison to only 21 out of 30 clusters created by MCLUST as shown in Figure 9(b). The median number of enriched categories for PUMA-CLUST and MCLUST are 7 and 3, respectively, as shown in Figure 10(b). Based on these GO enrichment analyses, it is evident that PUMA-CLUST generated more biologically relevant clusters than MCLUST.

**Figure 11:**
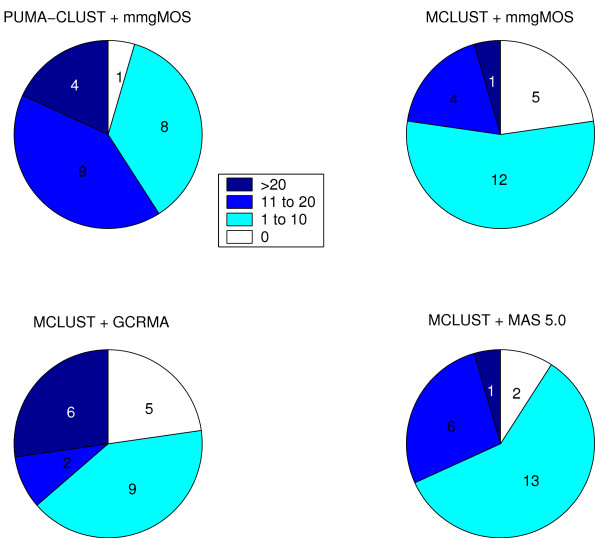
**Comparison of the number of clusters found with the indicated ranges of enriched GO categories for MCLUST and PUMA-CLUST clusters using various probe-level methods**. Comparison of the number of clusters found with the indicated ranges of enriched categories for MCLUST and PUMA-CLUST clusters using various probe-level methods when K = 22. For all comparisons, the enriched categories were found using GO Biological Process term level 5, enrichment cutoff at p-value of 0.05, and mouse (*Mus Musculus*) as the population background.

**Figure 12:**
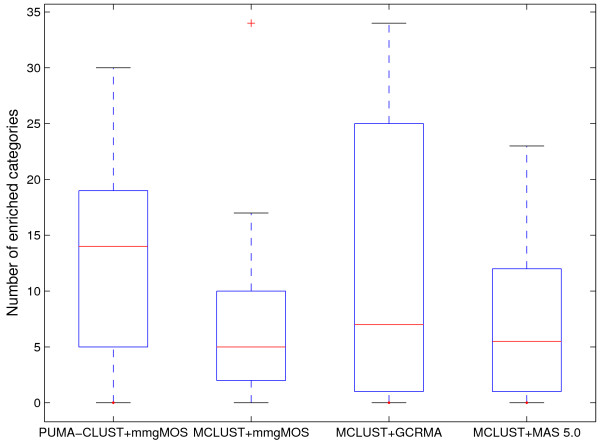
**Boxplot of the number of enriched categories for MCLUST and PUMA-CLUST clusters using various probe-level methods**. Boxplot of the number of enriched categories for MCLUST and PUMA-CLUST clusters using various probe-level methods when K = 22. The boxes show the lower quartile, median, and upper quartile values. The dotted lines show the extent of the rest of the data.

For further validation of the performance of PUMA-CLUST, we also applied MCLUST on multi-mgMOS measurements so that we can compare PUMA-CLUST with MCLUST using exactly the same probe-level summary method. MAS 5.0 is another popular probe-level method and therefore we also applied MCLUST to MAS 5.0 processed data for comparison. Enrichment analyses on the 22-cluster results for all four approaches (Figure 11 and Figure 12) show that MCLUST on multi-mgMOS processed data performed similarly to MCLUST on GCRMA processed data. Both have five clusters without any enriched category, but MCLUST with GCRMA had slightly higher median value for the number of enriched categories (7 vs. 5). Although MCLUST with MAS5.0 only had two clusters without any enriched category, its median value for the number of enriched categories is notably less than that of PUMA-CLUST with multi-mgMOS (5.5 vs. 14). Thus, PUMA-CLUST with multi-mgMOS still performs best in comparison to MCLUST using the three different expression summary methods. For 30-cluster results and for results with other numbers of clusters we found similar results. In particular, when the same probe-level method, multi-mgMOS, is used, PUMA-CLUST always outperforms MCLUST. The improved performance is due to the inclusion of the probe-level measurement error which down-weights the effect of the noisy low expressed genes.

## Conclusion

In this paper we demonstrate the usefulness of the measurement error in model-based clustering of gene expression data. A standard Gaussian mixture model with an unequal volume spherical covariance matrix is augmented to incorporate probe-level measurement error obtained from Affymetrix microarrays. Results from simulated datasets and a real mouse time-course dataset show that the inclusion of probe-level measurement error results in improved and more biologically meaningful clustering of gene expression data. The augmented clustering model has been implemented in an R package, *pumaclust*, for public use of the method.

The improved performance of the augmented model has been shown in this paper. It is possible that further improvement can also be made by considering the replicate information where repeated measurements are available for time points. Clustering on repeated measurements has been considered by [12,23,25], but all of these approaches do not include the probe-level measurement error. Including both probe-level noise and replicate information in the clustering would be a useful extension of our work.

## Methods

### multi-mgMOS and probe-level measurement error

Affymetrix microarrays use multiple probe-pairs called a probe-set to measure the expression level for each gene. Each probe-pair consists of a perfect match (PM) probe and a mismatch (MM) probe. By design, the intensity of the PM probe measures the specific hybridisation of the target and the MM probe measures the non-specific hybridisation associated to its corresponding PM probe. The microarray experimental data show that the intensities of both PM and MM probes vary in a probe-specific way and MM probes also detect some specific hybridisation. Based on these observations, multi-mgMOS [18] assumes the intensities of PM and MM probes for a probe-set both follow gamma distributions with parameters accounting for specific and non-specific hybridisation, and probe-specific effects. Let *y*_*ijc*_and *m*_*ijc *_represent the *j*th PM and MM intensities respectively for the *i*th probe-set under the *c*th condition. The model is defined by

*y*_*ijc *_~ Ga(*a*_*ic *_+ *α*_*ic*_, *b*_*ij*_)

*m*_*ijc *_~ Ga (*a*_*ic *_+ *φα*_*ic*_, *b*_*ij*_)     (5)

*b*_*ij *_~ Ga(*c*_*i*_, *d*_*i*_),

where Ga represents the gamma distribution. The parameter *a*_*ic *_accounts for the background and non-specific hybridisation associated with the probe-set and *α*_*ic *_accounts for the specific hybridisation measured by the probe-set. The parameter *b*_*ij *_is a latent variable which models probe-specific effects. The Maximum a Posteriori (MAP) solution of this model can be found by efficient numerical optimisation. The posterior distribution of the logged gene expression level can then be estimated from the model and approximated by a Gaussian distribution with a mean, x^
 MathType@MTEF@5@5@+=feaafiart1ev1aaatCvAUfKttLearuWrP9MDH5MBPbIqV92AaeXatLxBI9gBaebbnrfifHhDYfgasaacH8akY=wiFfYdH8Gipec8Eeeu0xXdbba9frFj0=OqFfea0dXdd9vqai=hGuQ8kuc9pgc9s8qqaq=dirpe0xb9q8qiLsFr0=vr0=vr0dc8meaabaqaciaacaGaaeqabaqabeGadaaakeaacuWG4baEgaqcaaaa@2E35@_*ic*_, and a variance, *ν*_*ic *_. The mean of this distribution is taken as the estimated gene expression for gene *i *under the condition *c *and the variance can be considered the measurement error associated with this estimate. The Gaussian approximation to the posterior distribution is useful for propagating the probe-level measurement error in subsequent downstream analyses.

### Mixture model

The mixture model is a useful tool for revealing the inherent structure of data. In a mixture model with *K *components, the data is generated by

p(xi)=∑k=1KP(k)p(xi|k;θk),     (6)
 MathType@MTEF@5@5@+=feaafiart1ev1aaatCvAUfKttLearuWrP9MDH5MBPbIqV92AaeXatLxBI9gBaebbnrfifHhDYfgasaacH8akY=wiFfYdH8Gipec8Eeeu0xXdbba9frFj0=OqFfea0dXdd9vqai=hGuQ8kuc9pgc9s8qqaq=dirpe0xb9q8qiLsFr0=vr0=vr0dc8meaabaqaciaacaGaaeqabaqabeGadaaakeaacqWGWbaCcqGGOaakieWacqWF4baEdaWgaaWcbaGaemyAaKgabeaakiabcMcaPiabg2da9maaqahabaGaemiuaaLaeiikaGIaem4AaSMaeiykaKIaemiCaaNaeiikaGIae8hEaG3aaSbaaSqaaiabdMgaPbqabaGccqGG8baFcqWGRbWAcqGG7aWoiiWacqGF4oqCdaWgaaWcbaGaem4AaSgabeaakiabcMcaPaWcbaGaem4AaSMaeyypa0JaeGymaedabaGaem4saSeaniabggHiLdGccqGGSaalcaWLjaGaaCzcamaabmaabaGaeGOnaydacaGLOaGaayzkaaaaaa@50D0@

where *P*(*k*) denotes the probability of selecting the *k*th component with parameters ***θ***_*k *_and ***θ ***= {*θ*_1_, ***θ***_2_,..., ***θ***_*K*_, *P*(*k*)} is the complete parameter set of the mixture model. The parameters *k *ar latent variables determining which cluster the data belongs to.

Mixture models are usually solved by maximum likelihood using an Expectation-Maximisation (EM) algorithm [34]. With the initialised parameters at *t *= 0, the values of parameters can be determined iteratively through an E-step and M-step:

• E-step: Compute

*P*^*t*^(*k*|***x***_*i*_) = *P*(*k*|*x*_*i*_; ***θ***^*t*^)     (7)

for each data point ***x***_*i *_and each component *k*.

• M-step:

θt+1=arg⁡max⁡θ∑i∑kPt(k|xi)log⁡(p(xi|k;θk)P(k))     (8)
 MathType@MTEF@5@5@+=feaafiart1ev1aaatCvAUfKttLearuWrP9MDH5MBPbIqV92AaeXatLxBI9gBaebbnrfifHhDYfgasaacH8akY=wiFfYdH8Gipec8Eeeu0xXdbba9frFj0=OqFfea0dXdd9vqai=hGuQ8kuc9pgc9s8qqaq=dirpe0xb9q8qiLsFr0=vr0=vr0dc8meaabaqaciaacaGaaeqabaqabeGadaaakeaaiiWacqWF4oqCdaahaaWcbeqaaiabdsha0jabgUcaRiabigdaXaaakiabg2da9iGbcggaHjabckhaYjabcEgaNnaaxababaGagiyBa0MaeiyyaeMaeiiEaGhaleaacqWF4oqCaeqaaOWaaabuaeaadaaeqbqaaiabdcfaqnaaCaaaleqabaGaemiDaqhaaOGaeiikaGIaem4AaSMaeiiFaWhcbmGae4hEaG3aaSbaaSqaaiabdMgaPbqabaGccqGGPaqkcyGGSbaBcqGGVbWBcqGGNbWzcqGGOaakcqWGWbaCcqGGOaakcqGF4baEdaWgaaWcbaGaemyAaKgabeaakiabcYha8jabdUgaRjabcUda7iab=H7aXnaaBaaaleaacqWGRbWAaeqaaOGaeiykaKIaemiuaaLaeiikaGIaem4AaSMaeiykaKIaeiykaKcaleaacqWGRbWAaeqaniabggHiLdGccaWLjaGaaCzcamaabmaabaGaeGioaGdacaGLOaGaayzkaaaaleaacqWGPbqAaeqaniabggHiLdaaaa@69A7@

with constraint ∑_*k *_*P*(*k*) = 1.

### Standard Gaussian mixture model

For mixture component distributions from the exponential family, like the Gaussian, both steps are exactly tractable. In a Gaussian mixture model where ***θ***_*k *_= {***μ***_*k*_, Σ_*k*_}, each component *k *is modelled by a Gaussian distribution with mean ***μ***_*k *_and covariance matrix Σ_*k*_,

p(xi|k;θk)=N(xi|μk,Σk)=1(2π)p|Σk|exp⁡(−12(xi−μk)TΣk−1(xi−μk),     (9)
 MathType@MTEF@5@5@+=feaafiart1ev1aaatCvAUfKttLearuWrP9MDH5MBPbIqV92AaeXatLxBI9gBaebbnrfifHhDYfgasaacH8akY=wiFfYdH8Gipec8Eeeu0xXdbba9frFj0=OqFfea0dXdd9vqai=hGuQ8kuc9pgc9s8qqaq=dirpe0xb9q8qiLsFr0=vr0=vr0dc8meaabaqaciaacaGaaeqabaqabeGadaaakeaafaqaaeGadaaabaGaemiCaaNaeiikaGccbmGae8hEaG3aaSbaaSqaaiabdMgaPbqabaGccqGG8baFcqWGRbWAcqGG7aWoiiWacqGF4oqCdaWgaaWcbaGaem4AaSgabeaakiabcMcaPaqaaiabg2da9aqaamrtHrhAL1wy0L2yHvtyaeHbnfgDOvwBHrxAJfwnaGabaiab91q8ojabcIcaOiab=Hha4naaBaaaleaacqWGPbqAaeqaaOGaeiiFaWNae4hVd02aaSbaaSqaaiabdUgaRbqabaGccqGGSaalcqqHJoWudaWgaaWcbaGaem4AaSgabeaakiabcMcaPaqaaaqaaiabg2da9aqaamaalaaabaGaeGymaedabaWaaOaaaeaacqGGOaakcqaIYaGmiiGacqaFapaCcqGGPaqkdaahaaWcbeqaaiabdchaWbaakmaaemaabaGaeu4Odm1aaSbaaSqaaiabdUgaRbqabaaakiaawEa7caGLiWoaaSqabaaaaOGagiyzauMaeiiEaGNaeiiCaa3aaeWaaeaacqGHsisldaWcaaqaaiabigdaXaqaaiabikdaYaaacqGGOaakcqWF4baEdaWgaaWcbaGaemyAaKgabeaakiabgkHiTiab+X7aTnaaBaaaleaacqWGRbWAaeqaaOGaeiykaKYaaWbaaSqabeaacqWGubavaaGccqqHJoWudaqhaaWcbaGaem4AaSgabaGaeyOeI0IaeGymaedaaOGaeiikaGIae8hEaG3aaSbaaSqaaiabdMgaPbqabaGccqGHsislcqGF8oqBdaWgaaWcbaGaem4AaSgabeaaaOGaayjkaiaawMcaaiabcYcaSaaacaWLjaGaaCzcamaabmaabaGaeGyoaKdacaGLOaGaayzkaaaaaa@872F@

where |·| denotes determinant and *p *is the dimension of the data. As well as changing the number of components in the mixture, the covariance matrix Σ_*k *_can be constrained to determine the flexibility of the model. The most constrained model is parameterised by Σ_*k *_= *σ*^2^*I *with only one free parameter in the covariance matrix for all components. The unconstrained model has full rank Σ_*k *_with *p*(*p *+ l)/2 free parameters in the covariance matrix for each component where *p *is the data dimension. All representations of the covariance matrix are explored in [35]. Allowing the number of free parameters in the covariance matrix to vary leads to various models accommodating varying characteristics of data. All of these models have been implemented in MCLUST [20] and the BIC model selection criterion (described later) is used to select the most appropriate model.

### Including measurement uncertainty in a Gaussian mixture model

From a probabilistic probe-level model, such as multi-mgMOS, for each data point one can obtain the measurement error, ***ν***_*i*_, which is a vector giving the variance of the measured expression level on each chip. Suppose ***x***_*i *_is the true expression level for data point *i*. The *k*th component of the Gaussian mixture model is modelled by *p*(***x***_*i*_|*k*; ***θ***_*k*_) = N
 MathType@MTEF@5@5@+=feaafiart1ev1aaatCvAUfKttLearuWrP9MDH5MBPbIqV92AaeXatLxBI9gBamrtHrhAL1wy0L2yHvtyaeHbnfgDOvwBHrxAJfwnaebbnrfifHhDYfgasaacH8akY=wiFfYdH8Gipec8Eeeu0xXdbba9frFj0=OqFfea0dXdd9vqai=hGuQ8kuc9pgc9s8qqaq=dirpe0xb9q8qiLsFr0=vr0=vr0dc8meaabaqaciaacaGaaeqabaWaaeGaeaaakeaaimaacqWFneVtaaa@383B@(***x***_*i*_|***μ***_*k*_, Σ_*k*_). The measured expression level x^
 MathType@MTEF@5@5@+=feaafiart1ev1aaatCvAUfKttLearuWrP9MDH5MBPbIqV92AaeXatLxBI9gBaebbnrfifHhDYfgasaacH8akY=wiFfYdH8Gipec8Eeeu0xXdbba9frFj0=OqFfea0dXdd9vqai=hGuQ8kuc9pgc9s8qqaq=dirpe0xb9q8qiLsFr0=vr0=vr0dc8meaabaqaciaacaGaaeqabaqabeGadaaakeaaieWacuWF4baEgaqcaaaa@2E3D@_*i *_can be expressed as x^
 MathType@MTEF@5@5@+=feaafiart1ev1aaatCvAUfKttLearuWrP9MDH5MBPbIqV92AaeXatLxBI9gBaebbnrfifHhDYfgasaacH8akY=wiFfYdH8Gipec8Eeeu0xXdbba9frFj0=OqFfea0dXdd9vqai=hGuQ8kuc9pgc9s8qqaq=dirpe0xb9q8qiLsFr0=vr0=vr0dc8meaabaqaciaacaGaaeqabaqabeGadaaakeaaieWacuWF4baEgaqcaaaa@2E3D@_*i *_= ***x***_*i *_+ ***ε***_*i*_. A zero-mean Gaussian measurement noise is assumed, ***ε***_*i *_~ N
 MathType@MTEF@5@5@+=feaafiart1ev1aaatCvAUfKttLearuWrP9MDH5MBPbIqV92AaeXatLxBI9gBamrtHrhAL1wy0L2yHvtyaeHbnfgDOvwBHrxAJfwnaebbnrfifHhDYfgasaacH8akY=wiFfYdH8Gipec8Eeeu0xXdbba9frFj0=OqFfea0dXdd9vqai=hGuQ8kuc9pgc9s8qqaq=dirpe0xb9q8qiLsFr0=vr0=vr0dc8meaabaqaciaacaGaaeqabaWaaeGaeaaakeaaimaacqWFneVtaaa@383B@(0, diag(***ν***_*i*_)), where diag(***ν***_*i*_) represents the diagonal matrix whose diagonal entries starting in the upper left corner are the elements of ***ν***_*i*_. Since x^
 MathType@MTEF@5@5@+=feaafiart1ev1aaatCvAUfKttLearuWrP9MDH5MBPbIqV92AaeXatLxBI9gBaebbnrfifHhDYfgasaacH8akY=wiFfYdH8Gipec8Eeeu0xXdbba9frFj0=OqFfea0dXdd9vqai=hGuQ8kuc9pgc9s8qqaq=dirpe0xb9q8qiLsFr0=vr0=vr0dc8meaabaqaciaacaGaaeqabaqabeGadaaakeaaieWacuWF4baEgaqcaaaa@2E3D@_*i *_is a linear sum of ***x***_*i *_and ***ε***_*i*_, the *k*th Gaussian component can be augmented as

*p*(x^
 MathType@MTEF@5@5@+=feaafiart1ev1aaatCvAUfKttLearuWrP9MDH5MBPbIqV92AaeXatLxBI9gBaebbnrfifHhDYfgasaacH8akY=wiFfYdH8Gipec8Eeeu0xXdbba9frFj0=OqFfea0dXdd9vqai=hGuQ8kuc9pgc9s8qqaq=dirpe0xb9q8qiLsFr0=vr0=vr0dc8meaabaqaciaacaGaaeqabaqabeGadaaakeaaieWacuWF4baEgaqcaaaa@2E3D@_*i*_|*k*; ***θ***_*k*_) = N
 MathType@MTEF@5@5@+=feaafiart1ev1aaatCvAUfKttLearuWrP9MDH5MBPbIqV92AaeXatLxBI9gBamrtHrhAL1wy0L2yHvtyaeHbnfgDOvwBHrxAJfwnaebbnrfifHhDYfgasaacH8akY=wiFfYdH8Gipec8Eeeu0xXdbba9frFj0=OqFfea0dXdd9vqai=hGuQ8kuc9pgc9s8qqaq=dirpe0xb9q8qiLsFr0=vr0=vr0dc8meaabaqaciaacaGaaeqabaWaaeGaeaaakeaaimaacqWFneVtaaa@383B@(x^
 MathType@MTEF@5@5@+=feaafiart1ev1aaatCvAUfKttLearuWrP9MDH5MBPbIqV92AaeXatLxBI9gBaebbnrfifHhDYfgasaacH8akY=wiFfYdH8Gipec8Eeeu0xXdbba9frFj0=OqFfea0dXdd9vqai=hGuQ8kuc9pgc9s8qqaq=dirpe0xb9q8qiLsFr0=vr0=vr0dc8meaabaqaciaacaGaaeqabaqabeGadaaakeaaieWacuWF4baEgaqcaaaa@2E3D@_*i*_|***μ***_*k*_, Σ_*k *_+ diag(***ν***_*i*_))     (10)

We therefore augment the mixture model to account for the measurement error of each data point,

p(xi)=∑k=1KP(k)N(xi|k;μk,Σk+diag(νi)).     (11)
 MathType@MTEF@5@5@+=feaafiart1ev1aaatCvAUfKttLearuWrP9MDH5MBPbIqV92AaeXatLxBI9gBaebbnrfifHhDYfgasaacH8akY=wiFfYdH8Gipec8Eeeu0xXdbba9frFj0=OqFfea0dXdd9vqai=hGuQ8kuc9pgc9s8qqaq=dirpe0xb9q8qiLsFr0=vr0=vr0dc8meaabaqaciaacaGaaeqabaqabeGadaaakeaacqWGWbaCcqGGOaakieWacqWF4baEdaWgaaWcbaGaemyAaKgabeaakiabcMcaPiabg2da9maaqahabaGaemiuaaLaeiikaGIaem4AaSMaeiykaKYenfgDOvwBHrxAJfwnHbqeg0uy0HwzTfgDPnwy1aaceaGae4xdX7KaeiikaGIae8hEaG3aaSbaaSqaaiabdMgaPbqabaGccqGG8baFcqWGRbWAcqGG7aWoiiWacqqF8oqBdaWgaaWcbaGaem4AaSgabeaakiabcYcaSiabfo6atnaaBaaaleaacqWGRbWAaeqaaOGaey4kaSIaeeizaqMaeeyAaKMaeeyyaeMaee4zaCMaeiikaGIae0xVd42aaSbaaSqaaiabdMgaPbqabaGccqGGPaqkcqGGPaqkaSqaaiabdUgaRjabg2da9iabigdaXaqaaiabdUealbqdcqGHris5aOGaeiOla4IaaCzcaiaaxMaadaqadaqaaiabigdaXiabigdaXaGaayjkaiaawMcaaaaa@6AF2@

Ideally, the covariance matrix should be of full rank to obtain the largest flexibility of the model. However, this will increase the complexity of the model. Since in (11) the additive measurement error diag(*ν*_*i*_) accounts for inherent variability in the data, especially for extremely noisy gene expression data, the unequal volume spherical model (VI) described in [10] with the covariance Σ_*k *_= σk2
 MathType@MTEF@5@5@+=feaafiart1ev1aaatCvAUfKttLearuWrP9MDH5MBPbIqV92AaeXatLxBI9gBaebbnrfifHhDYfgasaacH8akY=wiFfYdH8Gipec8Eeeu0xXdbba9frFj0=OqFfea0dXdd9vqai=hGuQ8kuc9pgc9s8qqaq=dirpe0xb9q8qiLsFr0=vr0=vr0dc8meaabaqaciaacaGaaeqabaqabeGadaaakeaaiiGacqWFdpWCdaqhaaWcbaGaem4AaSgabaGaeGOmaidaaaaa@30F4@*I *is adopted. This model allows the spherical components to have different variances which accounts for the variability within different gene function groups. Therefore, in this model the gene-specific variance ***ν***_*i *_is known and obtained from a probabilistic probe-level analysis model and the function-specific variance σk2
 MathType@MTEF@5@5@+=feaafiart1ev1aaatCvAUfKttLearuWrP9MDH5MBPbIqV92AaeXatLxBI9gBaebbnrfifHhDYfgasaacH8akY=wiFfYdH8Gipec8Eeeu0xXdbba9frFj0=OqFfea0dXdd9vqai=hGuQ8kuc9pgc9s8qqaq=dirpe0xb9q8qiLsFr0=vr0=vr0dc8meaabaqaciaacaGaaeqabaqabeGadaaakeaaiiGacqWFdpWCdaqhaaWcbaGaem4AaSgabaGaeGOmaidaaaaa@30F4@ is to be estimated from the mixture model via the EM algorithm. The parameters are denoted ***θ***_*k *_= {***μ***_*k*_, σk2
 MathType@MTEF@5@5@+=feaafiart1ev1aaatCvAUfKttLearuWrP9MDH5MBPbIqV92AaeXatLxBI9gBaebbnrfifHhDYfgasaacH8akY=wiFfYdH8Gipec8Eeeu0xXdbba9frFj0=OqFfea0dXdd9vqai=hGuQ8kuc9pgc9s8qqaq=dirpe0xb9q8qiLsFr0=vr0=vr0dc8meaabaqaciaacaGaaeqabaqabeGadaaakeaaiiGacqWFdpWCdaqhaaWcbaGaem4AaSgabaGaeGOmaidaaaaa@30F4@} for Gaussian component *k *and ***θ ***= {***θ***_1_, ***θ***_2_,..., ***θ***_*k *_} for all components, where *K *is the number of components. Using the K-means algorithm, one can obtain the initial parameters ***θ***^0 ^for all components. Equal probability of the component prior is also assumed for the initial value of *P*(*k*), *P*^0^(*k*). At the E-step, for each data point *x*_*i *_the posterior probability of belonging to component *k *is calculated as,

Pt(k|xi)=Pt(k|xi;θt−1)=P(xi|θkt−1)Pt−1(k)∑kP(xi|θkt−1)Pt−1(k).     (12)
 MathType@MTEF@5@5@+=feaafiart1ev1aaatCvAUfKttLearuWrP9MDH5MBPbIqV92AaeXatLxBI9gBaebbnrfifHhDYfgasaacH8akY=wiFfYdH8Gipec8Eeeu0xXdbba9frFj0=OqFfea0dXdd9vqai=hGuQ8kuc9pgc9s8qqaq=dirpe0xb9q8qiLsFr0=vr0=vr0dc8meaabaqaciaacaGaaeqabaqabeGadaaakeaafaqaaeGadaaabaGaemiuaa1aaWbaaSqabeaacqWG0baDaaGccqGGOaakcqWGRbWAcqGG8baFieWacqWF4baEdaWgaaWcbaGaemyAaKgabeaakiabcMcaPaqaaiabg2da9aqaaiabdcfaqnaaCaaaleqabaGaemiDaqhaaOGaeiikaGIaem4AaSMaeiiFaWNae8hEaG3aaSbaaSqaaiabdMgaPbqabaGccqGG7aWoiiWacqGF4oqCdaahaaWcbeqaaiabdsha0jabgkHiTiabigdaXaaakiabcMcaPaqaaaqaaiabg2da9aqaamaalaaabaGaemiuaaLaeiikaGIae8hEaG3aaSbaaSqaaiabdMgaPbqabaGccqGG8baFcqGF4oqCdaqhaaWcbaGaem4AaSgabaGaemiDaqNaeyOeI0IaeGymaedaaOGaeiykaKIaemiuaa1aaWbaaSqabeaacqWG0baDcqGHsislcqaIXaqmaaGccqGGOaakcqWGRbWAcqGGPaqkaeaadaaeqaqaaiabdcfaqjabcIcaOiab=Hha4naaBaaaleaacqWGPbqAaeqaaOGaeiiFaWNae4hUde3aa0baaSqaaiabdUgaRbqaaiabdsha0jabgkHiTiabigdaXaaakiabcMcaPiabdcfaqnaaCaaaleqabaGaemiDaqNaeyOeI0IaeGymaedaaOGaeiikaGIaem4AaSMaeiykaKcaleaacqWGRbWAaeqaniabggHiLdaaaOGaeiOla4caaiaaxMaacaWLjaWaaeWaaeaacqaIXaqmcqaIYaGmaiaawIcacaGLPaaaaaa@7E3D@

At the M-step, the component prior and the parameters of components are optimised,

Pt(k)=1N∑n=1NPt(k|xi)     (13)
 MathType@MTEF@5@5@+=feaafiart1ev1aaatCvAUfKttLearuWrP9MDH5MBPbIqV92AaeXatLxBI9gBaebbnrfifHhDYfgasaacH8akY=wiFfYdH8Gipec8Eeeu0xXdbba9frFj0=OqFfea0dXdd9vqai=hGuQ8kuc9pgc9s8qqaq=dirpe0xb9q8qiLsFr0=vr0=vr0dc8meaabaqaciaacaGaaeqabaqabeGadaaakeaafaqabeqadaaabaGaemiuaa1aaWbaaSqabeaacqWG0baDaaGccqGGOaakcqWGRbWAcqGGPaqkaeaacqGH9aqpaeaadaWcaaqaaiabigdaXaqaaiabd6eaobaadaaeWbqaaiabdcfaqnaaCaaaleqabaGaemiDaqhaaOGaeiikaGIaem4AaSMaeiiFaWhcbmGae8hEaG3aaSbaaSqaaiabdMgaPbqabaGccqGGPaqkaSqaaiabd6gaUjabg2da9iabigdaXaqaaiabd6eaobqdcqGHris5aaaakiaaxMaacaWLjaWaaeWaaeaacqaIXaqmcqaIZaWmaiaawIcacaGLPaaaaaa@4BB9@

$\begin{array}{ccc} \theta^{t} & = & \arg\underset{\theta }{\max}\sum_{i}\sum_{k}P^{t}(k|x_{i})\log(p(x_{i}|\theta_{k})P^{t}(k)) \end{array}.\;\left(14\right)$

Equation (14) cannot be solved analytically due to the incorporation of ***ν***_*i *_in the variance terms. However, with fast optimisation methods available such as SNOPT [36] and donlp2 [37], it is easy to calculate the optimal parameters numerically at the M-step. In our R implementation, *pumaclust*, we use donlp2.

### Model selection

In the previous section the covariance matrix of the Gaussian mixture model is specified and the parameters are worked out via an EM algorithm for a given *K*. In practice the most appropriate number of clusters should also be determined. In mixture models, the Bayesian Information Criterion (BIC [31]) is usually used to decide the appropriate number of clusters. For model *m *with the number of clusters *K*, the calculation of BIC is

BIC_*m *_= -2log *p*(*D*|θ^
 MathType@MTEF@5@5@+=feaafiart1ev1aaatCvAUfKttLearuWrP9MDH5MBPbIqV92AaeXatLxBI9gBaebbnrfifHhDYfgasaacH8akY=wiFfYdH8Gipec8Eeeu0xXdbba9frFj0=OqFfea0dXdd9vqai=hGuQ8kuc9pgc9s8qqaq=dirpe0xb9q8qiLsFr0=vr0=vr0dc8meaabaqaciaacaGaaeqabaqabeGadaaakeaaiiWacuWF4oqCgaqcaaaa@2E7A@_*m*_) + *d*_*m *_log(*n*),     (15)

where *D *is the dataset, *d*_*m *_is the number of free parameters to be estimated in model *m*, *n *is the number of genes and θ^
 MathType@MTEF@5@5@+=feaafiart1ev1aaatCvAUfKttLearuWrP9MDH5MBPbIqV92AaeXatLxBI9gBaebbnrfifHhDYfgasaacH8akY=wiFfYdH8Gipec8Eeeu0xXdbba9frFj0=OqFfea0dXdd9vqai=hGuQ8kuc9pgc9s8qqaq=dirpe0xb9q8qiLsFr0=vr0=vr0dc8meaabaqaciaacaGaaeqabaqabeGadaaakeaaiiWacuWF4oqCgaqcaaaa@2E7A@_*m *_are the estimated maximum likelihood parameters obtained by the EM algorithm. For the unequal volume spherical model (VI), the number of free parameters is *d*_*m *_= *K*(*p *+ 2) - 1. MCLUST also uses BIC to select the most appropriate class of covariance model.

### Adjusted rand index

The adjusted Rand index gives a measure of agreement between clustering results. Given a set of *n *data points *D *= {*x*_1_,..., *x*_*n*_}, suppose *C*^1 ^= {c11
 MathType@MTEF@5@5@+=feaafiart1ev1aaatCvAUfKttLearuWrP9MDH5MBPbIqV92AaeXatLxBI9gBaebbnrfifHhDYfgasaacH8akY=wiFfYdH8Gipec8Eeeu0xXdbba9frFj0=OqFfea0dXdd9vqai=hGuQ8kuc9pgc9s8qqaq=dirpe0xb9q8qiLsFr0=vr0=vr0dc8meaabaqaciaacaGaaeqabaqabeGadaaakeaacqWGJbWydaqhaaWcbaGaeGymaedabaGaeGymaedaaaaa@3008@,..., cM1
 MathType@MTEF@5@5@+=feaafiart1ev1aaatCvAUfKttLearuWrP9MDH5MBPbIqV92AaeXatLxBI9gBaebbnrfifHhDYfgasaacH8akY=wiFfYdH8Gipec8Eeeu0xXdbba9frFj0=OqFfea0dXdd9vqai=hGuQ8kuc9pgc9s8qqaq=dirpe0xb9q8qiLsFr0=vr0=vr0dc8meaabaqaciaacaGaaeqabaqabeGadaaakeaacqWGJbWydaqhaaWcbaGaemyta0eabaGaeGymaedaaaaa@303B@} and *C*^2 ^= {c12
 MathType@MTEF@5@5@+=feaafiart1ev1aaatCvAUfKttLearuWrP9MDH5MBPbIqV92AaeXatLxBI9gBaebbnrfifHhDYfgasaacH8akY=wiFfYdH8Gipec8Eeeu0xXdbba9frFj0=OqFfea0dXdd9vqai=hGuQ8kuc9pgc9s8qqaq=dirpe0xb9q8qiLsFr0=vr0=vr0dc8meaabaqaciaacaGaaeqabaqabeGadaaakeaacqWGJbWydaqhaaWcbaGaeGymaedabaGaeGOmaidaaaaa@300A@,..., cN2
 MathType@MTEF@5@5@+=feaafiart1ev1aaatCvAUfKttLearuWrP9MDH5MBPbIqV92AaeXatLxBI9gBaebbnrfifHhDYfgasaacH8akY=wiFfYdH8Gipec8Eeeu0xXdbba9frFj0=OqFfea0dXdd9vqai=hGuQ8kuc9pgc9s8qqaq=dirpe0xb9q8qiLsFr0=vr0=vr0dc8meaabaqaciaacaGaaeqabaqabeGadaaakeaacqWGJbWydaqhaaWcbaGaemOta4eabaGaeGOmaidaaaaa@303F@} represent two different partitions of the data points in *D*. Assume that *n*_*ij *_is the number of data points belonging to cluster ci1
 MathType@MTEF@5@5@+=feaafiart1ev1aaatCvAUfKttLearuWrP9MDH5MBPbIqV92AaeXatLxBI9gBaebbnrfifHhDYfgasaacH8akY=wiFfYdH8Gipec8Eeeu0xXdbba9frFj0=OqFfea0dXdd9vqai=hGuQ8kuc9pgc9s8qqaq=dirpe0xb9q8qiLsFr0=vr0=vr0dc8meaabaqaciaacaGaaeqabaqabeGadaaakeaacqWGJbWydaqhaaWcbaGaemyAaKgabaGaeGymaedaaaaa@3073@ and cj2
 MathType@MTEF@5@5@+=feaafiart1ev1aaatCvAUfKttLearuWrP9MDH5MBPbIqV92AaeXatLxBI9gBaebbnrfifHhDYfgasaacH8akY=wiFfYdH8Gipec8Eeeu0xXdbba9frFj0=OqFfea0dXdd9vqai=hGuQ8kuc9pgc9s8qqaq=dirpe0xb9q8qiLsFr0=vr0=vr0dc8meaabaqaciaacaGaaeqabaqabeGadaaakeaacqWGJbWydaqhaaWcbaGaemOAaOgabaGaeGOmaidaaaaa@3077@, and *n*_*i*_. and *n*._*j *_are the number of data points in cluster ci1
 MathType@MTEF@5@5@+=feaafiart1ev1aaatCvAUfKttLearuWrP9MDH5MBPbIqV92AaeXatLxBI9gBaebbnrfifHhDYfgasaacH8akY=wiFfYdH8Gipec8Eeeu0xXdbba9frFj0=OqFfea0dXdd9vqai=hGuQ8kuc9pgc9s8qqaq=dirpe0xb9q8qiLsFr0=vr0=vr0dc8meaabaqaciaacaGaaeqabaqabeGadaaakeaacqWGJbWydaqhaaWcbaGaemyAaKgabaGaeGymaedaaaaa@3073@ and cj2
 MathType@MTEF@5@5@+=feaafiart1ev1aaatCvAUfKttLearuWrP9MDH5MBPbIqV92AaeXatLxBI9gBaebbnrfifHhDYfgasaacH8akY=wiFfYdH8Gipec8Eeeu0xXdbba9frFj0=OqFfea0dXdd9vqai=hGuQ8kuc9pgc9s8qqaq=dirpe0xb9q8qiLsFr0=vr0=vr0dc8meaabaqaciaacaGaaeqabaqabeGadaaakeaacqWGJbWydaqhaaWcbaGaemOAaOgabaGaeGOmaidaaaaa@3077@ respectively. The adjusted Rand index can be calculated by

∑i,j(nij2)−[∑i(ni.2) ∑j(n.j2)]/(n2)12[∑i(ni.2)+∑j(n.j2)]−[∑i(ni.2)∑j(n.j2)]/(n2).     (16)
 MathType@MTEF@5@5@+=feaafiart1ev1aaatCvAUfKttLearuWrP9MDH5MBPbIqV92AaeXatLxBI9gBaebbnrfifHhDYfgasaacH8akY=wiFfYdH8Gipec8Eeeu0xXdbba9frFj0=OqFfea0dXdd9vqai=hGuQ8kuc9pgc9s8qqaq=dirpe0xb9q8qiLsFr0=vr0=vr0dc8meaabaqaciaacaGaaeqabaqabeGadaaakeaadaWcaaqaamaaqababaWaaeWaaeaafaqabeGabaaabaGaemOBa42aaSbaaSqaaiabdMgaPjabdQgaQbqabaaakeaacqaIYaGmaaaacaGLOaGaayzkaaaaleaacqWGPbqAcqGGSaalcqWGQbGAaeqaniabggHiLdGccqGHsisldaWadaqaamaaqababaWaaeWaaeaafaqabeGabaaabaGaemOBa42aaSbaaSqaaiabdMgaPbqabaGccqGGUaGlaeaacqaIYaGmaaaacaGLOaGaayzkaaaaleaacqWGPbqAaeqaniabggHiLdGccqqGGaaidaaeqaqaamaabmaabaqbaeqabiqaaaqaaiabd6gaUjabc6caUmaaBaaaleaacqWGQbGAaeqaaaGcbaGaeGOmaidaaaGaayjkaiaawMcaaaWcbaGaemOAaOgabeqdcqGHris5aaGccaGLBbGaayzxaaGaei4la8YaaeWaaeaafaqabeGabaaabaGaemOBa4gabaGaeGOmaidaaaGaayjkaiaawMcaaaqaamaalaaabaGaeGymaedabaGaeGOmaidaamaadmaabaWaaabeaeaadaqadaqaauaabeqaceaaaeaacqWGUbGBdaWgaaWcbaGaemyAaKgabeaakiabc6caUaqaaiabikdaYaaaaiaawIcacaGLPaaaaSqaaiabdMgaPbqab0GaeyyeIuoakiabgUcaRmaaqababaWaaeWaaeaafaqabeGabaaabaGaemOBa4MaeiOla4YaaSbaaSqaaiabdQgaQbqabaaakeaacqaIYaGmaaaacaGLOaGaayzkaaaaleaacqWGQbGAaeqaniabggHiLdaakiaawUfacaGLDbaacqGHsisldaWadaqaamaaqababaWaaeWaaeaafaqabeGabaaabaGaemOBa42aaSbaaSqaaiabdMgaPbqabaGccqGGUaGlaeaacqaIYaGmaaaacaGLOaGaayzkaaaaleaacqWGPbqAaeqaniabggHiLdGcdaaeqaqaamaabmaabaqbaeqabiqaaaqaaiabd6gaUjabc6caUmaaBaaaleaacqWGQbGAaeqaaaGcbaGaeGOmaidaaaGaayjkaiaawMcaaaWcbaGaemOAaOgabeqdcqGHris5aaGccaGLBbGaayzxaaGaei4la8YaaeWaaeaafaqabeGabaaabaGaemOBa4gabaGaeGOmaidaaaGaayjkaiaawMcaaaaacqGGUaGlcaWLjaGaaCzcamaabmaabaGaeGymaeJaeGOnaydacaGLOaGaayzkaaaaaa@8DB0@

## Authors' contributions

XL developed and implemented the new model, applied the new and standard models to the simulated and real data, and drafted the manuscript. KL and AB provided the mouse dataset, helped with the evaluation of the clustering results on this dataset and revised the manuscript. MR supervised the study and helped with the manuscript preparation. All authors read and approved the final manuscript.
